# Intertumoral Genetic Heterogeneity Generates Distinct Tumor Microenvironments in a Novel Murine Synchronous Melanoma Model

**DOI:** 10.3390/cancers13102293

**Published:** 2021-05-11

**Authors:** Shuyang S. Qin, Booyeon J. Han, Alyssa Williams, Katherine M. Jackson, Rachel Jewell, Alexander C. Chacon, Edith M. Lord, David C. Linehan, Minsoo Kim, Alexandre Reuben, Scott A. Gerber, Peter A. Prieto

**Affiliations:** 1 Department of Microbiology & Immunology, University of Rochester School of Medicine & Dentistry, University of Rochester, Rochester, NY 14642, USA; Shuyang_Qin@urmc.rochester.edu (S.S.Q.); Booyeon_Han@urmc.rochester.edu (B.J.H.); edith_lord@urmc.rochester.edu (E.M.L.); Minsoo_Kim@urmc.rochester.edu (M.K.); Scott_Gerber@urmc.rochester.edu (S.A.G.); 2 Center for Tumor Immunology Research, University of Rochester Medical Center, University of Rochester, Rochester, NY 14642, USA; Alyssa_Williams@urmc.rochester.edu (A.W.); Katherine_Jackson@urmc.rochester.edu (K.M.J.); Rachel_Jewell@urmc.rochester.edu (R.J.); Alexander_chacon@urmc.rochester.edu (A.C.C.); David_Linehan@urmc.rochester.edu (D.C.L.); 3 Department of Surgery, University of Rochester Medical Center, University of Rochester, Rochester, NY 14642, USA; 4 Department of Thoracic/Head & Neck Medical Oncology, The University of Texas MD Anderson Cancer Center, The University of Texas, Houston, TX 77030, USA; areuben@mdanderson.org

**Keywords:** tumor microenvironment, tumor heterogeneity, metastatic melanoma, tumor models, immunomodulation, PD-L1

## Abstract

**Simple Summary:**

Metastatic melanoma patients may present with multiple, simultaneous metastases that are genetically different. This intertumoral heterogeneity can cause these tumors to respond differently to the same systemic therapy. Progression of any one tumor, even when others regress, eventually leads to therapy termination. The mechanism underlying these mixed responses remains unknown due to a lack of clinically representative animal models. In a novel murine model of synchronous melanoma that recapitulates human intertumoral heterogeneity, we show that intertumoral genetic heterogeneity leads to the simultaneous generation of distinct tumor immune microenvironments within the same mouse. Furthermore, each tumor can independently regulate local PD-1 (programmed cell death protein 1) and PD-L1 (PD-1 ligand) expressions, an immunosuppressive axis targeted by popular checkpoint immunotherapies. This model is useful for furthering the study of intertumoral heterogeneity and of lesion-specific therapeutic responses.

**Abstract:**

Metastatic melanoma portends a poor prognosis and patients may present with multiple, simultaneous tumors. Despite recent advances in systemic immunotherapy, a majority of patients fail to respond, or exhibit lesion-specific responses wherein some metastases respond as others progress within the same patient. While intertumoral heterogeneity has been clinically associated with these mixed lesion-specific therapeutic responses, no clear mechanism has been identified, largely due to the scarcity of preclinical models. We developed a novel murine synchronous melanoma model that recapitulates this intertumoral genetic and microenvironmental heterogeneity. We show that genetic differences between tumors are sufficient to generate distinct tumor immune microenvironments (TIME) simultaneously in the same mouse. Furthermore, these TIMEs lead to the independent regulation of PD-1/PD-L1 (programmed cell death protein 1/PD-1 ligand), a popular axis targeted by immune checkpoint therapy, in response to ongoing anti-tumor immunity and the presence of interferon-gamma. Currently, therapeutic selection for metastatic melanoma patients is guided by a single biopsy, which may not represent the immune status of all tumors. As a result, patients can display heterogeneous lesion-specific responses. Further investigations into this synchronous melanoma model will provide mechanistic insight into the effects of intertumoral heterogeneity and guide therapeutic selection in this challenging patient population.

## 1. Introduction

Metastatic melanoma remains a deadly disease, with a median survival of less than 6 months [1]. Despite the curative potential of immunotherapies targeting the programmed cell death (PD)-1 pathway, less than 40% of stage IV melanoma patients respond, and even fewer achieve remission [2,3,4]. This variability is further complicated in synchronous metastatic melanoma, where multiple lesions are diagnosed within 6 months. The vast majority of these patients develop therapeutic resistance over time or lesion-specific mixed responses, wherein some metastases respond and others progress [3,5]. The current standard of care requires a single biopsy to dictate therapy selection, which may not be representative of all existing tumors [6]. This selection bias can negatively impact quality of life, as patients often suffer adverse events without therapeutic benefit. As such, novel methods are needed to personalize therapeutic selection for synchronous metastatic melanoma patients in order to generate optimal responses and minimize adverse events.

Whole exome sequencing of different metastatic lesions within the same patient demonstrates that melanoma metastases may share as little as 21% of somatic mutations [5]. Malignant melanoma has been identified as having one of the highest somatic mutational burdens, with a significant number of mutations being lesion-specific [7,8]. Intertumoral genetic heterogeneity has been clinically correlated to tumor immune microenvironmental (TIME) differences and subsequent lesion-specific therapeutic responses in multiple cancer types [5,9,10]. Specifically, PD-1 immunotherapy response has been positively correlated to increasing tumor expression of PD-L1 (PD-1 ligand) and the number of tumor-infiltrating CD8+ T cells [2,11,12]. Chronic inflammation and PD-L1 expression are thought to upregulate PD-1 on CD8+ T cells, inducing T cell exhaustion [13,14,15]. Exhausted T cells exhibit decreased proliferation and effector cytokine secretion, both of which could be potentially rescued by α-PD-1 immunotherapy [13,16,17]. One potent inducer of PD-L1 expression is interferon-gamma (IFN-γ), a critical cytokine in functional anti-tumor immune responses [18,19]. The mechanisms underlying how these different components converge to determine differential immunogenicity and immunotherapeutic responses in synchronous metastatic melanoma tumors are not well understood.

A major obstacle to studying the impact of intertumoral heterogeneity on anti-melanoma immunity is the scarcity of animal synchronous and metastatic models that recapitulate human disease. For instance, approximately 50–60% of melanoma patients have the *BRAF^V600E^* driver mutation, 60% have inactivating *CDKN2A* mutations and 5–20% have inactivating *PTEN* mutations [20]. The most commonly used mouse melanoma cell lines, including B16, harbor wildtype driver genes and thus cannot genetically represent the majority of human melanoma [21]. To remedy this, the Yale University Mouse Melanoma lines were developed with the YUMM 1.7 (YUMM) cell line containing the common *Braf^V600E/WT^, Pten^−/−^,* and *Cdkn2*^−/−^ driver mutations combination [22]. YUMMER 1.7 (YUMMER) is a more immunogenic cell line derived from YUMM after multiple rounds of ultraviolet-B (UVB) irradiation in order to simulate the most common mechanism for generating physiological mutations in melanoma [7,23,24]. YUMM and YUMMER cell lines share approximately 40% of somatic mutations [23]. Thus, these two cell lines are optimal candidates to model the observed intertumoral heterogeneity present in synchronous metastatic melanoma patients.

We developed a novel murine synchronous melanoma model using the YUMM and YUMMER cell lines that recapitulates the intertumoral heterogeneity observed in human synchronous metastatic melanoma patients. We found that the YUMM and YUMMER melanoma lines generate distinct TIMEs varying in tumor-infiltrating immune cell types and surface marker expressions associated with T cell checkpoints. Furthermore, we discovered that tumor genetics and the presence of IFN-γ differentially drive the local regulation of PD-1 and PD-L1 expression within each synchronous tumor. Thus, we propose a new preclinical model of intertumoral heterogeneity for synchronous melanoma that may be studied to uncover mechanisms underlying lesion-specific immune and immunotherapeutic responses.

## 2. Materials and Methods

### 2.1. In Vivo Animal Studies

All in vivo procedures were performed in accordance with the University of Rochester’s University Committee on Animal Resources approved guidelines. Six- to eight-week-old wildtype and age-matched *Ifng*^−/−^ (B6.129S7-*Ifng^tm1Ts^/*J) C57BL/6J mice were obtained from The Jackson Laboratory (Bar Harbor, ME, USA) and from a generous gift by Edith Lord, PhD. Animals were given at least one week to acclimate before establishment of subcutaneous tumors.

### 2.2. Cell Cultures

The YUMM 1.7 cell line was purchased from ATCC. The YUMMER 1.7 cell line was generously gifted by Dr. Marcus Bosenberg. Cell cultures were maintained in DMEM:F12 media (Gibco, Waltham, MA, USA) supplemented with 10% fetal bovine serum (Gibco), 1% penicillin/streptomycin (Thermo Fisher Scientific, Waltham, MA, USA) and 1% MEM non-essential amino acid solution (Gibco) at 37 °C and 5% CO_2_.

### 2.3. In Vitro Interferon-γ Studies

The YUMM1.7 and YUMMER 1.7 cell lines were plated in 6-well dishes and cultured in phosphate-buffered saline (PBS, Gibco) with 1 ng/mL, 10 ng/mL, or 100 ng/mL of mouse IFN-γ (R&D Systems, Minneapolis, MN, USA) in regular culture media. All cells within the well were collected at given time-points to either be counted for total live cell numbers following trypan blue staining or stained for surface markers in subsequent flow cytometric analyses.

### 2.4. Tumor Model and Tumor Volume Measurements

In total, 1 × 10^6^ YUMM 1.7 or YUMMER 1.7 cells were simultaneously injected subcutaneously into opposing flanks of C57BL/6J mice in 100 μL of PBS. Cell lines were detached with 0.25% trypsin/EDTA (Gibco) and resuspended in PBS (Gibco) for injection. Tumor growth was assessed with caliper measurements. Tumor volume was calculated by the formula $\frac{length\times width^{2}}{2}$.

### 2.5. Tumor Single-Cell Suspensions

Mice tumors were individually excised, mechanistically dissociated, and digested in an enzyme solution containing 10 mM HEPES (Gibco), 1 mg/mL Type IV Collagenase (Sigma-Aldrich, St. Louis, MO, USA), 150 U/mL Type IV DNase I (Sigma-Aldrich) and 2.5 U/mL Type V Hyaluronidase (Sigma-Aldrich) in RPMI (Gibco). Enzymatic digests were homogenized in the gentleMACS C tubes (Miltenyi Biotec, Auburn, CA, USA) by alternating three times between 30-s pulse dissociation with a gentleMACS dissociator (Miltenyi Biotec) and 10-min incubation at 37 °C. Homogenates were passed through 70 μm filters and cells were resuspended in staining buffer (1 mg/mL sodium azide and 10 mg/mL BSA in PBS) to a final concentration of approximately 1–2 × 10^6^ cells/100 μL.

### 2.6. Immunohistochemistry

Murine melanoma tumors were resected and paraffin-embedded. Tissue sections of 5 µm thickness were prepared using a cryostat (Leica, Buffalo Grove, IL, USA). Tissues were deparaffinized and rehydrated using serial EtOH dilutions. Tissue sections were incubated in H_2_O_2_ for 30 min at room temperature. Sections were stained with hematoxylin and counterstained with eosin. For IHC, rehydrated slides were incubated in blocking buffer (DAKO non-serum protein block, Agilent, Santa Clara, CA, USA) for an hour followed by primary CD45 antibody (1:1000 dilution, AF114, R&D Systems) incubation overnight. Sections were stained with horseradish peroxidase-labeled secondary antibody (1:200 dilution, BA-9500, Vector Laboratories, Burlingame, CA, USA) for 30 min followed by DAB application (DAKO, Agilent). Slides were counterstained with hematoxylin. Slides were washed three times with PBS between each stain. Slides were imaged using the Olympus DP80 (Center Valley, PA, USA) imaging system.

### 2.7. Flow Cytometry

The following conjugated antibodies were used for flow cytometric staining: PerCP/Cy5.5 anti-mouse CD45 (30-F11, BD Biosciences, San Jose, CA, USA), FITC anti-mouse F4/80 (CI:A3-1, Abcam, Cambridge MA, USA), APC/Cy7 anti-mouse CD8a (53.67, Thermo Fisher Scientific), APC/Cy5.5 anti-mouse CD4 (GK1.5, Southern Biotech, Birmingham, AL, USA), APC anti-mouse PD-L1 (10F.9G2, Biolegend, San Diego, CA, USA), BV786 anti-mouse CD11b (M1/70, Biolegend), BV711 anti-mouse CD103 (M290, BD Biosciences), BV605 anti-mouse CD19 (1D3, Biolegend), PB anti-mouse Ly6G (1A8, Biolegend), PE/Cy7 anti-mouse Ly6C (HK1.4, Biolegend), PE/Cy5 anti-mouse IA/IE (M5/114.15.2, Thermo Fisher Scientific), PE/CF594 anti-mouse NK1.1 (PK136, BD Biosciences), PE anti-mouse CD11c (N418, Thermo Fisher Scientific), FITC anti-mouse CD106 (429, Thermo Fisher Scientific), BV605 anti-mouse H-2Kb (AF6-88.5, BD Bioscience), PE anti-mouse CD119 (2E2, Thermo Fisher Scientific), BV421 anti-mouse IA/IE (M5/114.15.2, BD Biosciences), PE/Cy7 anti-mouse PD1 (RMP1-30, Biolegend), and PE/Cy5 anti-mouse CD3e (145-2C11, Biolegend). Cell surface antigens were stained for 30 min at 4 °C in the dark. Following two staining buffer washes, the cells were fixed with BD Cytofix (BD Biosciences) for 20 min at 4 °C in the dark before resuspension in staining buffer until analysis. Samples were run on a LSRII Fortessa (BD Biosciences). At least one hundred thousand events were collected per sample and analyzed using FlowJo software (BD Life Science, Franklin Lakes, NJ, USA).

### 2.8. Luminex Analyte Assay

Following sacrifice, mice tumors were individually excised and homogenized with a tissue homogenizer in 700 μL of Cell Lysis Buffer 2 (R&D Systems) containing 1x Halt Protease Inhibitor Cocktail (Thermo Fisher Scientific). Tissues were lysed on ice for 30 min with gentle agitation. Magnetic Luminex Assays were performed with a Mouse Premixed Cytokine/Chemokine Multi-Analyte Kit (R&D Systems) per manufacturer’s instructions. Microplates were run on a Bio-Flex 200 system (Bio-Rad, Hercules, CA, USA), collecting 50–100 beads per target with less than 20% aggregate. Pierce BCA protein assays (Thermo Fisher Scientific Waltham, MA, USA) were performed on the remaining lysates following the manufacturer’s instructions to determine total protein concentrations. Analyte concentrations were normalized to total protein concentration for each sample into pg analyte/mg protein.

### 2.9. RNA Sequencing and Analysis

Cells were detached from the culture with 0.25% trypsin and lysed in RLT Plus buffer (QIAGEN, Germantown, MD, USA) containing 1% β-mercaptoethanol. Lysates were homogenized with QIAShredder spin columns, and RNA was purified using the RNeasy Micro Kit (QIAGEN) following the manufacturer’s instructions. RNA sequencing and preliminary differentially expressed gene analysis were performed by the University of Rochester Genomics Research Center. RNA quality was assessed using an Agilent Bioanalyzer (Agilent) and cDNA libraries were constructed with TruSeq RNA Sample Preparation Kit V2 (Illumina, San Diego, CA, USA) according to the manufacturer’s instructions. Sequencing was performed on HiSeqTM 2500 (Illumina). Raw reads were demultiplexed using bcl2fastq version 2.19.1 and mapped to the Mus musculus reference genome (GRCm38 + Gencode-M22 Annotation) using STAR_2.7. Differential expression analysis was performed using DESeq2-1.22.1 with a P-value threshold of 0.05, within R version 3.5.1. Subsequent pathway analysis was performed using Ingenuity Pathway Analysis (IPA) software (QIAGEN, Germantown, MD, USA).

### 2.10. Quantification and Statistical Analyses

Prism 8 software (GraphPad, San Diego, CA, USA) was used for all statistical analyses with *p*-values < 0.05 determined to be statistically significant. Tumor growth data were analyzed by mixed-model analysis with Tukey’s multiple comparisons test at each time-point. Tumor rejection data were analyzed by the logrank test. All flow cytometry gating was performed using FlowJo 10 software (BD Life Science, Franklin Lakes, NJ, USA) with one-way ANOVA or paired *t*-test analyses to assess for statistically significant differences in cell density or geometric mean fluorescence intensity between various groups of tumors or cell lines. All diagrammatic figures were created with BioRender (Toronto, ON, Canada).

## 3. Results

### 3.1. YUMM and YUMMER Cell Lines Upregulate Different Immunomodulatory Pathways and Generate Synchronous Melanoma Tumors in the Same Mouse

**Figure 1:**
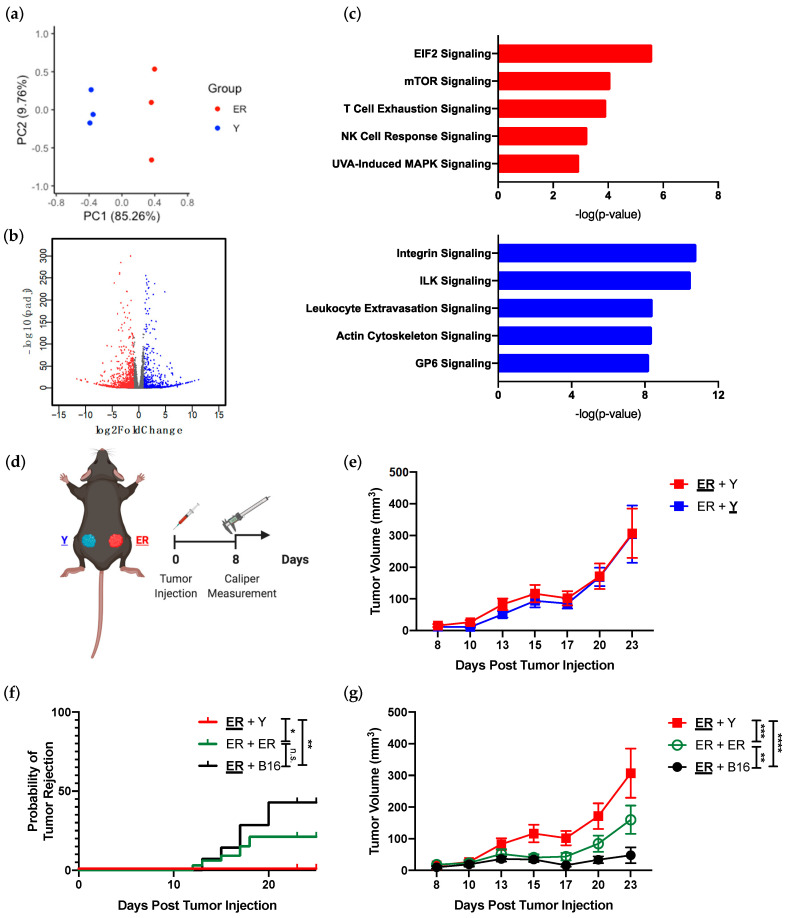
YUMMER (ER) and YUMM (Y) cell lines have different transcriptomes in vitro and establish synchronous melanoma tumors in vivo. (**a**) PCA plot separating overall transcriptomic differences between ER and Y cell lines in vitro. Notice how the two cell lines generate distinct groupings along the PC1 axis. (**b**) Volcano plot highlighting differentially expressed genes in ER (red) vs. Y (blue) cell lines. Colored genes are statistically significant with adjusted *p*-value < 0.05 and log 2-fold change > |1|. (**c**) Top-most upregulated pathways in ER and Y cell lines as identified by IPA analysis. Red bars indicate pathways upregulated in ER and blue bars indicate pathways upregulated in Y cells. (**d**) Synchronous murine melanoma model schematic. The analyzed tumor in the synchronous model is **underlined and bolded** in subsequent figures. A table of tumor combination can be found in Supplementary Table S1. (**e**) Growth curves of individual ER (**ER** + Y) and Y (ER + **Y**) tumors in synchronous melanoma mice. (**f**) Percent of ER tumors rejected and growth curve (**g**) of synchronous ER tumors from ER + Y, ER + ER, and ER + B16 mice. Data (mean ± SEM) in (**e**–**g**) are pooled, from 3–5 mice/group/experiment, and representative of at least 2 independent experiments. * *p* < 0.05, ** *p* < 0.01, *** *p* < 0.001, **** *p* < 0.0001, n.s. not significant.

We assessed the YUMM and YUMMER transcriptomes to determine if UVB radiation significantly altered gene expression in these cell lines. More than 6000 genes (6197) were differentially expressed between the two cell lines (Figure 1a,b). As whole exome sequencing revealed 1446 nonsynonymous exonic mutations (roughly 60%) between the two cell lines [23], UVB radiation is capable of inducing transcriptomic changes in addition to genomic mutations. Pathway analysis demonstrated that the YUMMER cell line upregulated pathways associated with DNA damage repair (EIF2 signaling) and UV-induced stress (UVA-induced MAPK signaling), which are congruent with the circumstances of its generation (Figure 1c). Interestingly, the most upregulated pathways in both YUMMER and YUMM lines include immunomodulatory pathways, such as T cell exhaustion and natural killer (NK) cell response signaling for YUMMER, as well as leukocyte extravasation signaling for YUMM (Figure 1c). Thus, these two melanoma cell lines are optimal for establishing an in vivo murine model of synchronous melanoma given their distinct transcriptomes, and similar proportions of shared and unique mutations as reported in previous analyses of human synchronous metastatic melanomas [5,23].

To generate a murine synchronous melanoma model, we simultaneously injected YUMMER and YUMM cells into the subcutaneous tissue of the left and right flanks of the same C57BL/6J mouse, respectively (Figure 1d and Supplementary Table S1). As these mice have two types of tumors present, the analyzed tumor will be **bolded and underlined**. Synchronous YUMM and YUMMER tumors displayed similar growth kinetics in vivo with an initial period of equilibrium followed by tumor escape after day 20 (Figure 1e). Interestingly, the presence of a contralateral YUMM tumor not only prevented the synchronous YUMMER tumor from spontaneous rejection, but also facilitated its growth (Figure 1f,g). This induction of YUMMER tumor growth was highest with the genetically heterogeneous YUMM tumor, compared to that with identical YUMMER tumor or with B16 melanoma tumor. Thus, we have established that the YUMMER and YUMM cell lines can be used to generate synchronous melanoma tumors in vivo. Furthermore, this combination of YUMMER and YUMM tumors exhibits differential growth from other synchronous melanoma tumor pairs without intertumoral genetic heterogeneity.

### 3.2. Synchronous YUMMER and YUMM Tumors Establish Immunologically Distinct Tumor Immune Microenvironments

**Figure 2:**
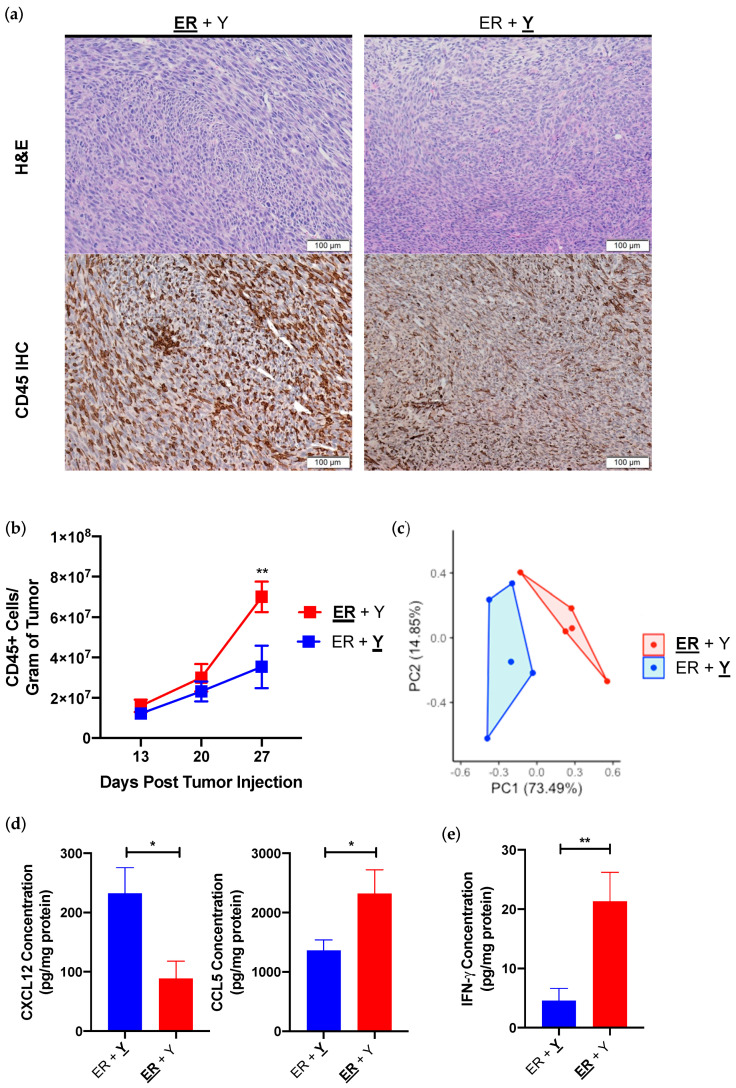
Synchronous YUMMER (ER) and YUMM (Y) tumors establish distinct TIME in vivo. (**a**) Representative H&E histology and CD45 IHC of synchronous ER (**ER** + Y) and Y (ER + **Y**) tumors on day 27. (**b**) Number of infiltrating CD45+ immune cells over time in synchronous ER (red) and Y (blue) tumors. N = 3–5 mice per time point. (**c**) PCA plot from Luminex analysis of 25 different chemokines/cytokines of synchronous ER and Y tumors on day 27. Concentrations of intratumoral CCL5, CXCL12 (**d**) and IFN-γ (**e**) in synchronous ER and Y tumors isolated from synchronous melanoma mice on day 27. Data (mean ± SEM) in (**c**–**e**) are pooled, from 2–3 mice/group/experiment, and are representative of at least 2 independent experiments. * *p* < 0.05, ** *p* < 0.01. H&E (hematoxylin and eosin); IHC (immunohistochemistry); IFN-γ (interferon-gamma).

We examined intratumoral immune cells since cell line transcriptome analysis suggested that YUMMER tumors may preferentially induce immune, or specifically lymphocytic, response over the YUMM tumors (Figure 1c). Both YUMMER and YUMM form fairly dense tumors in vivo with similar morphologies, with the exception of increased CD45+ immune infiltration in YUMMER tumors as detected by histology (Figure 2a). Fixed time-point analysis showed a steady increase in tumor-infiltrating CD45+ immune cells in YUMM tumors over time, whereas the frequency of tumor-infiltrating immune cells significantly increased in YUMMER tumors 27 days after tumor implantation (Figure 2b). As leukocyte migration is primarily driven by chemokine and cytokine gradients [25,26], we performed Luminex analyses on 25 common intratumoral chemokines and cytokines (Supplementary Figure S1). Synchronous YUMMER and YUMM tumors in the same mouse contain distinct intratumoral chemokine/cytokine profiles (Figure 2c), including YUMM overexpression of CXCL12 and YUMMER overexpression of CCL5 (Figure 2d). The in vivo elevation of these two cytokines is likely secondary to cell line genetics as YUMM and YUMMER cell lines also upregulated corresponding mRNA levels in vitro (Supplementary Figure S2). One cytokine that is differentially expressed in vivo but is undetectable in either cell line in vitro is IFN-γ, which is significantly elevated in YUMMER tumors (Figure 2e).

**Figure 3:**
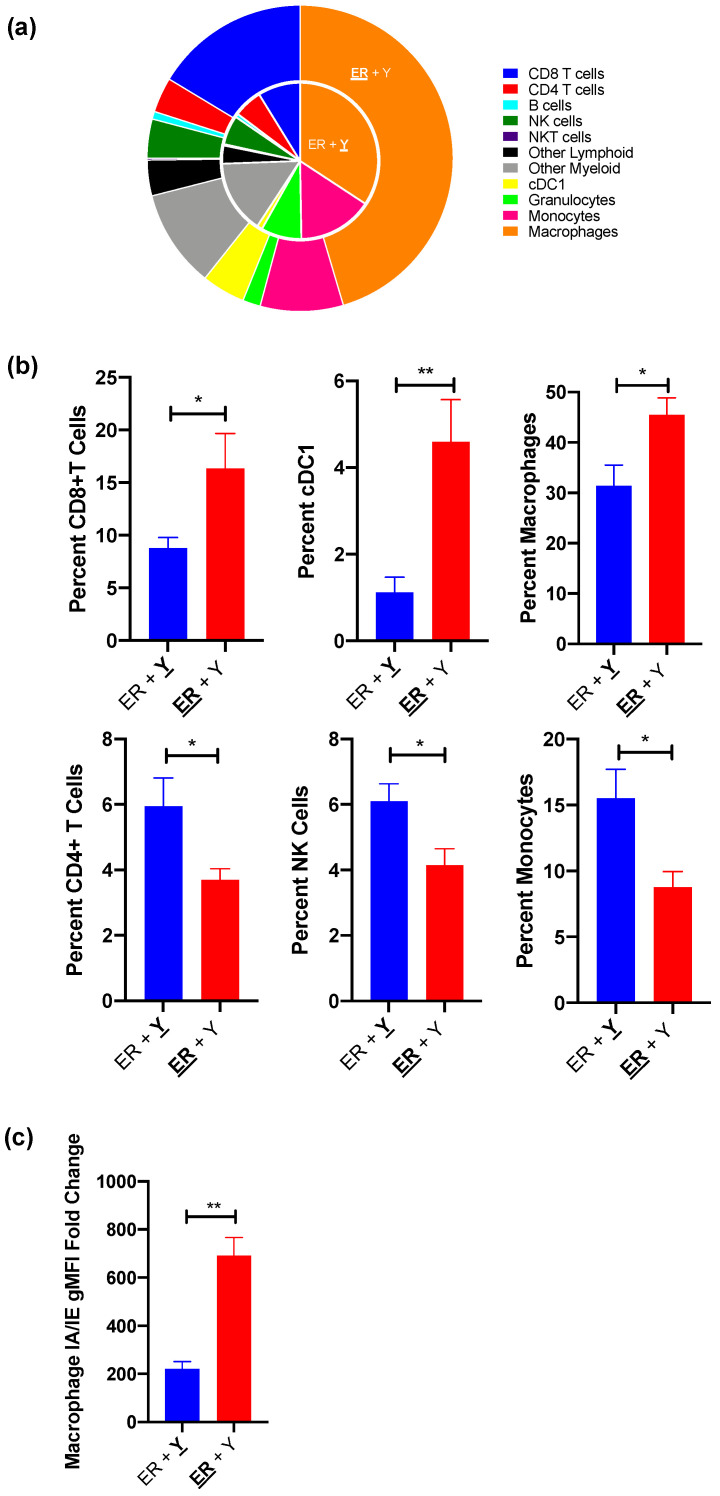
Synchronous YUMMER (ER) and YUMM (Y) TIMEs contain different immune cell infiltrations by day 27 post tumor implantation. (**a**) Nested pie charts demonstrating distributions of various CD45+ immune cell subsets in synchronous ER (**ER** + Y) and Y (ER + **Y**) tumors with Y tumor as the inside pie and ER tumor the outside pie. (**b**) Synchronous ER (red) tumors have increased percentages of CD8+ T cell, cDC1 and macrophage infiltration, whereas synchronous Y (blue) tumors have increased CD4+ T cell, NK cell and monocyte infiltration. (**c**) The geometric mean fluorescence intensity (gMFI) of the surface MHC Class II I-A/I-E molecule is higher in synchronous ER tumors compared to Y tumors after normalization to unstained cells. Data (mean ± SEM) in (**a**,**b**) are pooled from 3–5 mice/group/experiment and representative of at least two independent experiments. The gating schematic for (**b**) is shown in Supplementary Figure S3. Data (mean ± SEM) in (**c**) are pooled from 3–5 mice/group. * *p* < 0.05, ** *p* < 0.01. TIME (tumor immune microenvironment); NK (natural killer); cDC1 (dendritic cell type 1); MHC (major histocompatibility complex).

As IFN-γ is a critical cytokine that shapes tumor development and has both pro-tumorigenic and anti-tumorigenic properties [19,27,28], we analyzed the immune infiltration within synchronous YUMMER and YUMM tumors to determine the anti-tumor immunological activation status of each TIME. Of the nine quantified subsets, the percentages of six cell types are significantly altered between simultaneously present YUMMER and YUMM TIMEs (Figure 3a). Synchronous YUMMER tumors have increased infiltrations of macrophages along with CD8+ T cells and cross-presenting cDC1 cells, the latter two being commonly associated with strong anti-tumor immune responses (Figure 3b). Furthermore, YUMMER-infiltrating macrophages have increased surface I-A/I-E expression (Figure 3c), a marker indicative of a more activated phenotype [29,30,31], compared to those infiltrating the contralateral YUMM tumors. In contrast, YUMM tumors have increased infiltrations of CD4+ T cells, NK cells, and monocytes. Both the intratumoral immune cell type distribution and the chemokine/cytokine profile suggest that despite being on opposite flanks of the same mouse, YUMMER and YUMM tumors establish distinct TIMEs, with the former being more immunogenic.

### 3.3. Immunological Differences in YUMM and YUMMER TIMEs Are Amplified by Their Response to IFN-γ

**Figure 4:**
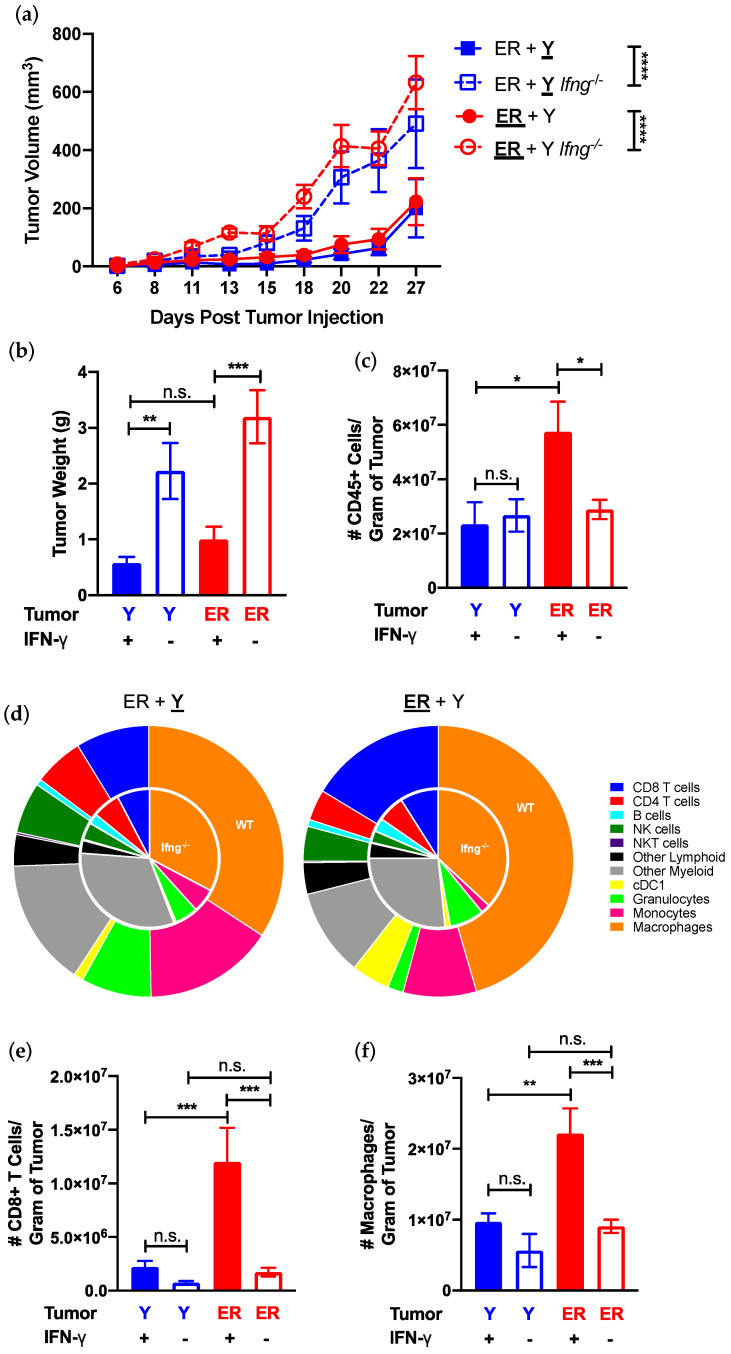
IFN-γ presence influences synchronous YUMMER (ER) and YUMM (Y) tumor growth and establishment of TIME. (**a**) Growth curves of individual ER (**ER** + Y, red) and Y (ER + **Y**, blue) tumors in the synchronous melanoma model in wildtype and *Ifng*^−/−^ mice. (**b**) Tumor weight and (**c**) frequency of tumor-infiltrating CD45+ immune cells per gram of tumor assessed on day 27. (**d**) Immune cell type distribution in synchronous ER (**ER** + Y) and Y (ER + **Y**) tumors shown as nested pie charts with inner pie representing percentages found in *Ifng*^−/−^ mice and outer pie representing wildtype mice. Frequency of tumor-infiltrating CD8+ T cells (**e**) and macrophages (**f**) on day 27 of individual tumors in wildtype and *Ifng*^−/−^ synchronous mice. Data (mean ± SEM) in (**a**,**b**) are pooled, from 3–8 mice/group from two independent experiments. Data (mean ± SEM) in (**c**–**f**) are pooled from 3–5 mice/group/experiment and representative of at least two independent experiments. Gating schematic for (**d**) shown in Supplementary Figure S3. * *p* < 0.05, ** *p* < 0.01, *** *p* < 0.001, **** *p* < 0.0001, n.s. not significant. TIME (tumor immune microenvironment); IFN-γ (interferon-gamma); NK (natural killer); cDC1 (dendritic cell type 1).

As one of the few differentially expressed effector cytokines in synchronous YUMM and YUMMER tumors (Figure 2d), we investigated the immunomodulatory effects of IFN-γ in our model. Synchronous YUMM and YUMMER tumors grew faster and larger in *Ifng*^−/−^ than wildtype mice (Figure 4a,b), indicating that the growth of both tumors is restricted by IFN-γ. Further investigations into the individual TIMEs revealed that the lack of IFN- γ abolished the increase in CD45+ leukocytes observed in synchronous YUMMER tumors, but had no effect on the number of immune cells in the YUMM tumors (Figure 4c). The distribution of various immune subsets between *Ifng*^−/−^ synchronous YUMM and YUMMER tumors was comparable and similar to that of wildtype synchronous YUMM tumors (Figure 4d). In contrast, the presence of IFN-γ resulted in a preferential increase in the number of CD8+ T cells and macrophages in YUMMER tumors (Figure 4e,f and Supplementary Figure S4a,b).

**Figure 5:**
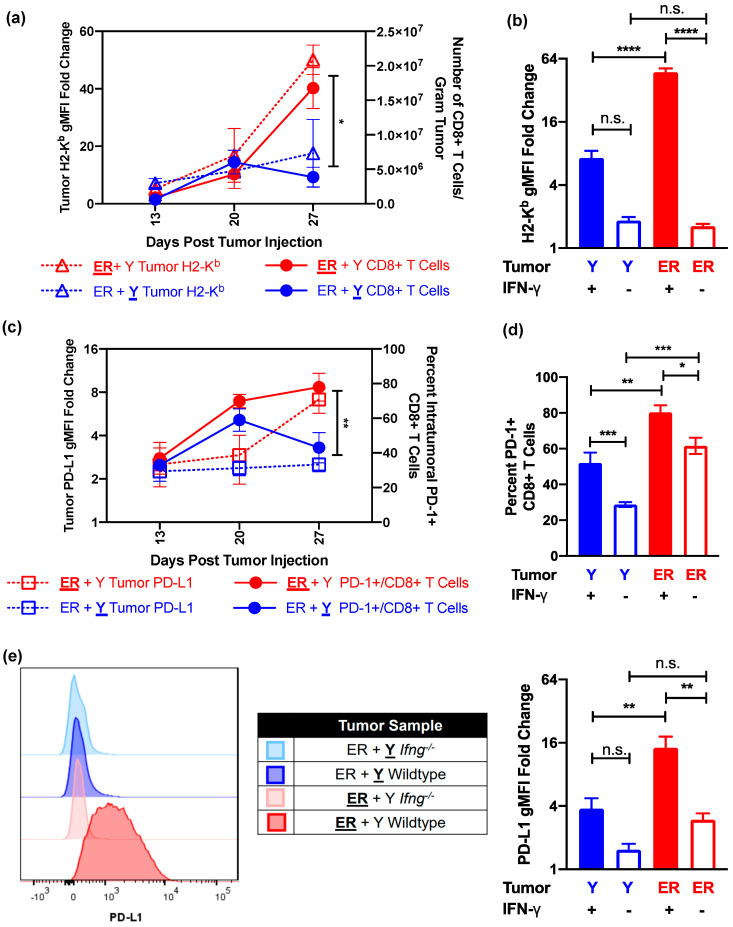
IFN-γ presence modulates local H2-K^b^ expression and PD-1/PD-L1 axis in synchronous YUMMER (ER) and YUMM (Y) tumors. (**a**) Relationship between tumor surface H2-K^b^ and recruited CD8+ T cells over time in synchronous ER (**ER** + Y, red) and Y (ER + **Y**, blue) tumors. (**b**) Geometric MFI of surface H2-K^b^, normalized to unstained cells, on tumor cells 27 days after tumor implantation in wildtype and *Ifng*^−/−^ mice. (**c**) Relationship between tumor surface PD-L1 and the percentage of intratumoral PD-1+ CD8+ T cells over time. (**d**) Percent of PD-1+ CD8+ T cells on day 27 in wildtype and *Ifng*^−/−^ synchronous mice. (**e**) Representative flow plots and quantification of normalized geometric MFI of surface PD-L1 on tumor cells 27 days after implantation in wildtype and *Ifng*^−/−^ mice. Data (mean ± SEM) in (**a**,**c**) are derived from 3–5 mice per time point. Data (mean ± SEM) in (**b**,**d**,**e**) are pooled from 3–5 mice/group/experiment and representative of at least two independent experiments. * *p* < 0.05, ** *p*< 0.01, *** *p* < 0.001, **** *p* < 0.0001, n.s. not significant. IFN-γ (interferon-gamma); H2-K^b^ (murine major histocompatibility complex class I); PD-1 (programmed cell death protein 1); PD-L1 (PD-1 ligand); MFI (mean fluorescence intensity).

CD8+ T cell-mediated anti-tumor immunity is dependent on the presence of major histocompatibility complex (MHC) class I molecules, which in turn can be upregulated by IFN-γ [18,32,33,34,35,36]. Both YUMMER and YUMM cells have the capacity to similarly upregulate the surface MHC Class I molecule H2-K^b^ in response to exogenous IFN-γ in vitro (Supplementary Figure S5a). The increase in the frequency of YUMMER-infiltrating CD8+ T cells over time is directly proportional to the induction of tumor surface H2-K^b^ (Figure 5a), whereas contralateral YUMM tumors fail to upregulate surface H2-K^b^ and elicit additional CD8+ T cells. However, as YUMMER cells have elevated *H2-K1* mRNA expression at the baseline compared to YUMM cells (Supplementary Figure S2), we wanted to determine if H2-K^b^ upregulation is cell line-intrinsic or secondary to the presence of intratumoral IFN-γ. Not only do YUMMER tumors in *Ifng*^−/−^ mice fail to upregulate surface H2-K^b^, but they also express it at a level similar to contralateral YUMM tumors (Figure 5b). These data, coupled with significantly reduced CD8+ T cell infiltration in *Ifng*^−/−^ mice, suggest that local IFN-γ concentration is a significant driver of the differential adaptive immune responses of the two tumors.

Thus far, we have shown that synchronous YUMMER tumors establish more immunologically activated TIMEs with increased CD8+ T cell infiltration than contralateral YUMM tumors in the same mouse. However, the two tumors display similar growth kinetics. To investigate why the anti-YUMMER immune reaction fails to control these tumors, we interrogated the PD-1/PD-L1 checkpoint, a popular axis involved in T cell exhaustion that is targeted by immune checkpoint therapy [4,37,38]. YUMMER tumors upregulate surface expressions of PD-L1 along with H2-K^b^ to induce and accumulate a high percentage of PD-1+ CD8+ T cells (Figure 5c). In contrast, the contralateral YUMM tumors do not upregulate surface PD-L1 nor retain elevated percentages of PD-1+ CD8+ T cells over time (Figure 5c). Furthermore, YUMMER tumors were unable to maintain elevated levels of intratumoral PD-1+ CD8+ T cells (Figure 5d) and surface PD-L1 expression (Figure 5e) without the presence of IFN-γ. Interestingly, while the percentage of tumor-infiltrating PD-1+ CD8+ T cells decreased in both YUMM and YUMMER tumors without IFN-γ, approximately 60% of YUMMER-infiltrating CD8+ T cells were still PD-1+ in *Ifng*^−/−^ mice (Figure 5d). To eliminate the possibility that YUMM tumors cannot upregulate PD-L1 expression, we incubated YUMM and YUMMER cell lines with exogenous IFN-γ in vitro, whereby both cell lines similarly upregulated surface PD-L1 expression (Supplementary Figure S5b). Overall, these data suggest that cell line genetics determine the establishment of different TIMEs, while the resulting intratumoral IFN-γ concentration maintains and intensifies these differences in synchronous melanoma tumors, as exemplified by the tumor-specific regulation of H2-K^b^ and PD-L1 expressions.

## 4. Discussion

Genomic instability is one of the main mechanisms behind the generation of intertumoral genetic heterogeneity in metastatic cancers [39]. UV-induced DNA damage accounts for the majority of somatic mutations in malignant melanoma, making it the cancer with the highest mutational burden [7,24]. One clinical study has identified lesion-specific mutations between melanoma metastases within the same patient and correlated this intertumoral difference to heterogeneous microenvironments or mixed-responses to systemic treatments [5]. However, the underlying mechanism has not been identified due to the lack of clinically relevant preclinical models. We have created a murine model of synchronous melanoma that recapitulates the intertumoral genetic and microenvironmental heterogeneity observed among patients. Using our model, we demonstrate that genetic differences between the YUMM and YUMMER cell lines are sufficient to alter the tumors’ responses to inflammation and to simultaneously establish distinct TIMEs on opposing flanks of the same mouse. These genetic and microenvironmental differences converge in the local regulation of MHC Class I expression and of the PD-1/ PD-L1 axis on tumor and tumor-infiltrating CD8+ T cells.

**Figure 6:**
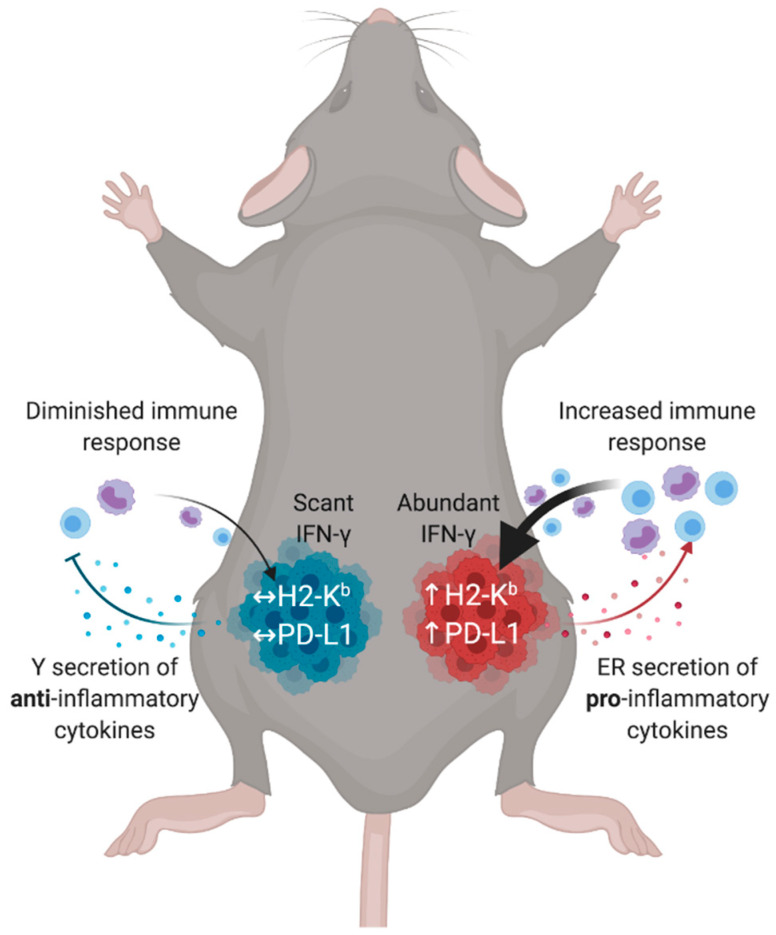
Tumor genetics and microenvironmental factors contribute to the generation of distinct TIMEs in synchronous melanoma. Cell line genetics determine the secretion chemokines and cytokines that influence initial immune infiltrations. These immune infiltrations then determine effector molecule gradients, such as IFN-γ. Immunologically hot tumors have abundant IFN-γ, leading to the regional tumor upregulation of surface molecules such as H2-K^b^ and PD-L1 that cement the TIME polarity and generate a feedback loop. In contrast, immunologically cold tumors cannot establish sufficient initial immune infiltration to activate the TIME. TIME (tumor immune microenvironment); IFN-γ (interferon-gamma); H2-K^b^ (murine major histocompatibility complex class I); PD-L1 (ligand for programmed cell death protein 1).

Genetic differences between the YUMM and YUMMER cell lines may establish a predilection toward leukocyte recruitment via cytokines/chemokines secretion or cell surface molecules expressions. For instance, YUMMER have increased mRNA levels of *H2-K1, H2-D1* (encoding MHC Class I molecules), *Cd80*, and *Ccl5*, whereas YUMM have increased mRNA levels of *Cxcl12* (Supplementary Figure S2). YUMM tumors potentially evade the immune system by increasing the production of CXCL12, whose high expression is associated with the chemo-repulsion of T cells and the exclusion of other CD45+ leukocyte infiltration [40,41]. In contrast, YUMMER tumors upregulate CCL5, a chemokine shown to recruit cross-presenting cDC1s into tumors [19,42], generating a microenvironment favorable to further augment lymphocytic recruitment. The presence of cDC1s induces increased T cell priming and migration [43,44], as demonstrated by the YUMMER TIMEs containing significantly higher numbers of intratumoral CD8+ T cells and intratumoral IFN-γ concentrations. Abundant IFN-γ, in turn, locally upregulate surface H2-K^b^ and PD-L1 expressions on tumor cells, creating a feedback loop that further cements the immunogenicity of the tumor and fully establishes the TIME (Figure 6).

Chronic inflammation and antigenic presentation, both conditions present in YUMMER tumors, have been associated with the induction of PD-1/PD-L1-mediated T cell exhaustion [13,45,46]. Although the scope of these experiments does not functionally assess the exhaustion status of intratumoral T cells, we have demonstrated that synchronous YUMMER tumors upregulate surface PD-L1 expression and accumulate PD-1+ CD8+ T cells, and that this process is dependent on the presence of IFN-γ. In contrast, in synchronous YUMM tumors with lower intratumoral IFN-γ levels and surface H2-K^b^ expression, the PD-1/PD-L1 pathway is less activated. Interestingly, the presence of the more immunologically active YUMMER tumor cannot elicit a stronger anti-tumor immune reaction to the contralateral YUMM tumor. However, inversely, the presence of the YUMM tumors can facilitate the growth of contralateral YUMMER tumors and prevent their rejection, a phenomenon that cannot be replicated with synchronous YUMMER or B16 tumors. These results indicate that while the genetic differences between YUMM and YUMMER tumors are sufficient to simultaneously establish two immunologically distinct microenvironments in the same mouse, the cross-talk between the tumors can potentially affect systemic anti-tumor immunity and promote immune evasion. Recent experiments have shown that anti-tumor effector T cells can migrate between two identical tumors and that antigen dose may determine the immunodominance of particular CD8+ T cell clones [47,48]. Given that the two YUMM and YUMMER tumors share a significant amount of synonymous somatic mutations, T cells recognizing shared neoantigens may become both immunodominant and preferentially exhausted in synchronous melanoma hosts. As a result, the overall pool of dysfunction T cells enlarges, eventually depleting systemic resources and leading to subsequent tumor immune escape. Understanding the systemic effects elicited by the presence of multiple, genetically heterogeneous tumors is crucial to improving metastatic therapy, and is a question that our synchronous model is poised to answer.

Given that therapeutic selection for metastatic melanoma patients often depends on one biopsy of a single tumor, the assessment of individual melanoma metastases may be necessary to more accurately classify the metastatic microenvironments for the optimization of therapy selection. As PD-1 inhibitors, such as nivolumab and pembrolizumab, become more widely used as first-line treatments for patients with metastatic melanoma, the need to identify predicative biomarkers intensified [4,49]. While tumor PD-L1 expression and intratumoral CD8+ T cell PD-1 expression are indicative of T cell exhaustion [37,46], their utility as immunotherapy biomarkers has so far been limited. However, our model demonstrates that despite deriving from the YUMM cell line, YUMMER cells differentially regulate their own PD-L1 expression. Given the divergent lesion-specific responses to systemic immunotherapy exhibited by synchronous metastatic melanoma patients, the heterogeneity in PD-L1 expression between tumors may be a better biomarker to predict overall therapeutic response.

Heterogeneous, or so called “mixed”, responses to systemic therapy prove to be an ongoing dilemma in therapeutic selection for metastatic patients, as disease progression in one of many lesions may lead to therapy termination. A clinically relevant murine model that can isolate lesion-specific effects is needed to effectively study the mechanisms of immunosuppression and therapeutic resistance. We present a preclinical model that verifies that select genetic differences between synchronously present melanoma metastases can generate distinct TIMEs poised for immune evasion. This model is well-suited to answer questions linking tumor genetics to tumor immunology in vivo, and to identify potential targets to overcome lesion-specific therapeutic resistances in synchronous metastatic melanoma. Further investigations of intertumoral heterogeneity will guide the selection of effective combinatory therapies that cover all immunosuppressive mechanisms present in all melanoma metastases.

## 5. Conclusions

Metastatic melanoma patients who present with multiple, synchronous metastases often exhibit lesion-specific responses to systemic immunotherapy [5]. The progression of even one out of many metastases may lead to therapy termination despite the regression of other tumor lesions within the same patient. The optimization of therapy selection requires knowledge of potential underlying lesion-specific therapeutic resistance mechanisms, which have largely remained unexplored due to a lack of representative preclinical models. We have generated a novel murine model of synchronous melanoma that recapitulates the clinically observed genetic and microenvironmental heterogeneity using the YUMM and YUMMER cell lines, which share approximately 60% of somatic mutations. We demonstrate that these genetic differences lead to the simultaneous establishment of immunologically distinct tumor microenvironments in the same mouse. Furthermore, these tumors can differentially regulate the PD-1 checkpoint axis in response to ongoing immune response and IFN-γ presence. This preclinical model is thus poised to investigate mechanisms of immunotherapy resistance in synchronous melanoma as the usage of PD-1 inhibitors for the treatment of metastatic melanoma becomes more widespread.

## Data Availability

Data that support the findings of this study are included in the article or uploaded as online supplemental information.

## Supplementary Materials

The following are available online at https://www.mdpi.com/article/10.3390/cancers13102293/s1, Table S1: Tumor legend notation, Figure S1: Heatmap of intratumoral cytokines/chemokines, Figure S2: Heatmap of key differentially expressed genes in Y vs. ER cells in vitro, Figure S3: Gating scheme for flow cytometric analysis of tumor-infiltrating immune subsets, Figure S4: Percentages of tumor-infiltrating CD8+ T cells and macrophages in synchronous Y and ER tumors of wildtype and *Ifng*^−/−^ mice, Figure S5: Normalized geometric MFI of surface H2-K^b^ and PD-L1 on Y and ER cells cultured with exogenous IFN-γ in vitro.
