# Supplementary material for: Intertumoral Genetic Heterogeneity Generates Distinct Tumor Microenvironments in a Novel Murine Synchronous Melanoma Model
Source: Cancers (Basel). 2021 May 11;13(10):2293. doi: 10.3390/cancers13102293 (PMC8151632; doi:10.3390/cancers13102293)
Supplement: Supplementary file 1 [file cancers-13-02293-s001.zip › cancers-1159458-supplementary.pdf]

Supplementary Materials:

Name	Tumor Analyzed	Tumor Combinations in Mice
**ER** + Y	YUMMER	**YUMMER** + YUMM
ER + **Y**	YUMM	YUMMER + **YUMM**
ER + ER	YUMMER	YUMMER + YUMMER
**ER** + B16	YUMMER	**YUMMER** + B16

Table S1: Tumor legend notation. YUMM (Y); YUMMER (ER)

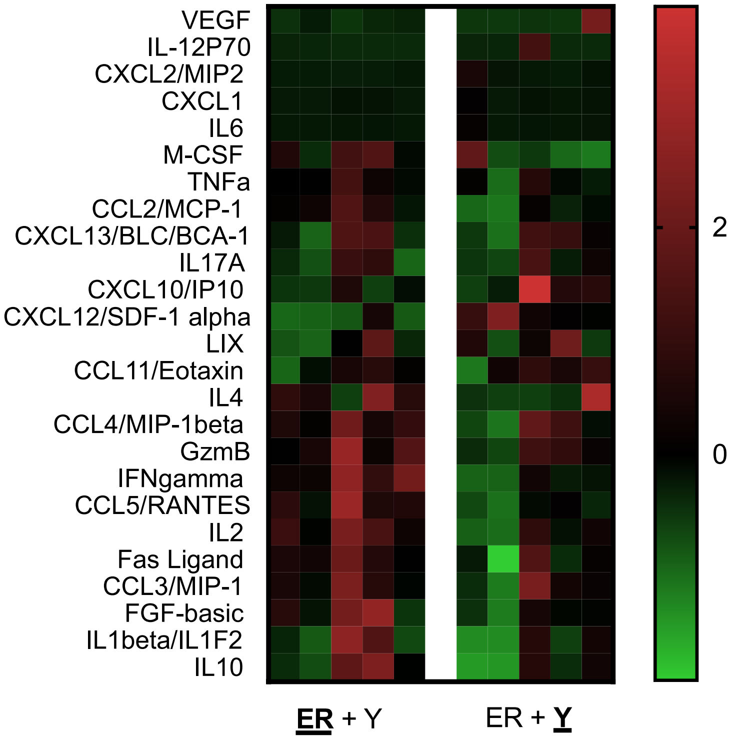

Figure S1: Heatmap of intratumoral cytokines/chemokines in synchronous ER (**ER** + Y) and Y (ER + **Y**) tumors harvested on day 27 as determined by Luminex analysis.

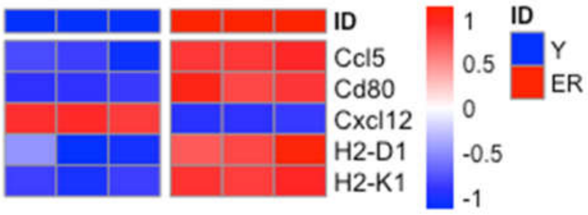

Figure S2: Heatmap of key differentially expressed genes in YUMM (Y) vs. YUMMER (ER) cells in vitro.

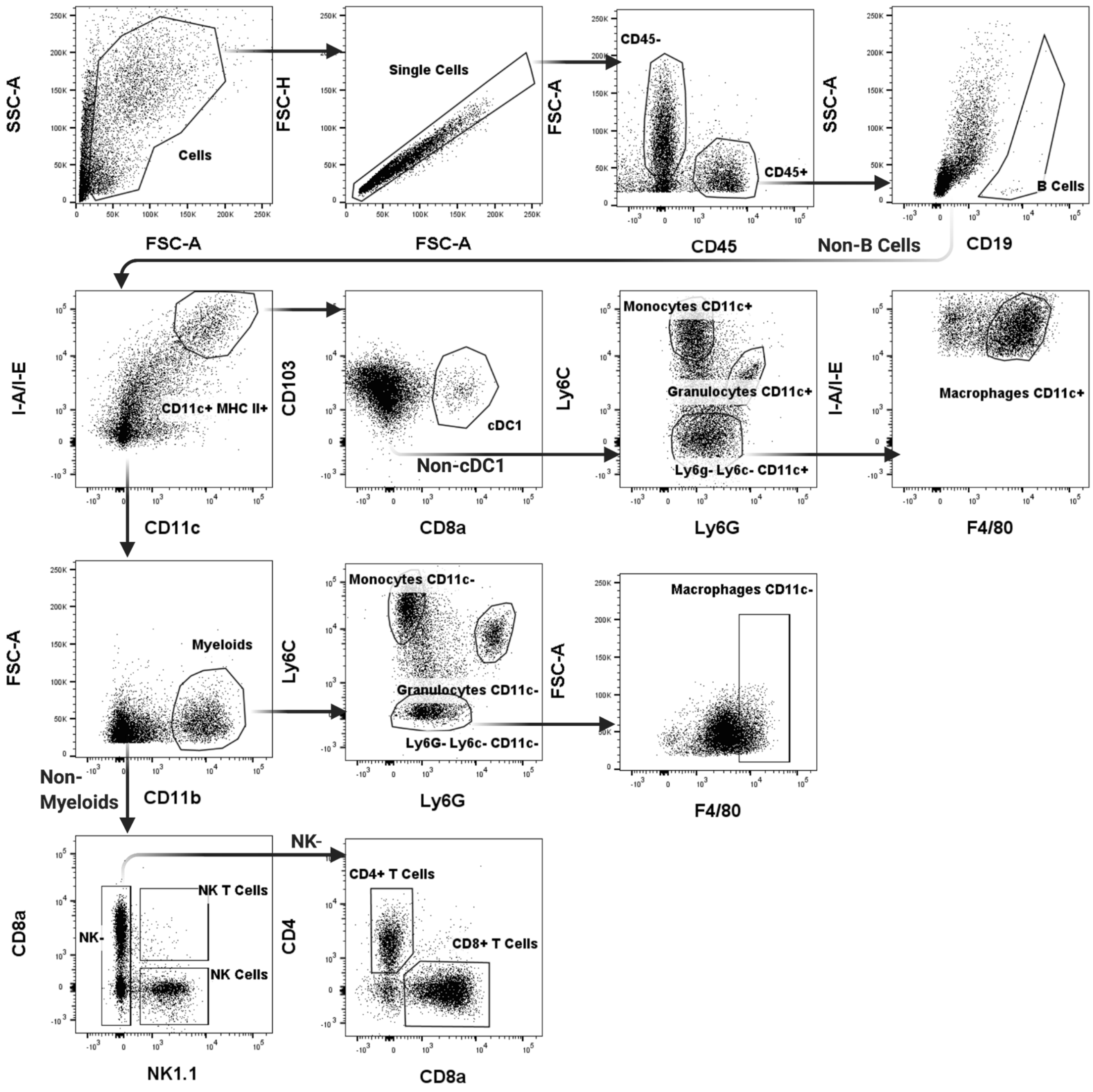

Figure S3: The gating scheme for flow cytometric analysis of tumor-infiltrating immune subsets. Total macrophages, monocytes and granulocytes refer to the concatenated sum of respective CD11c+ and CD11c- populations.

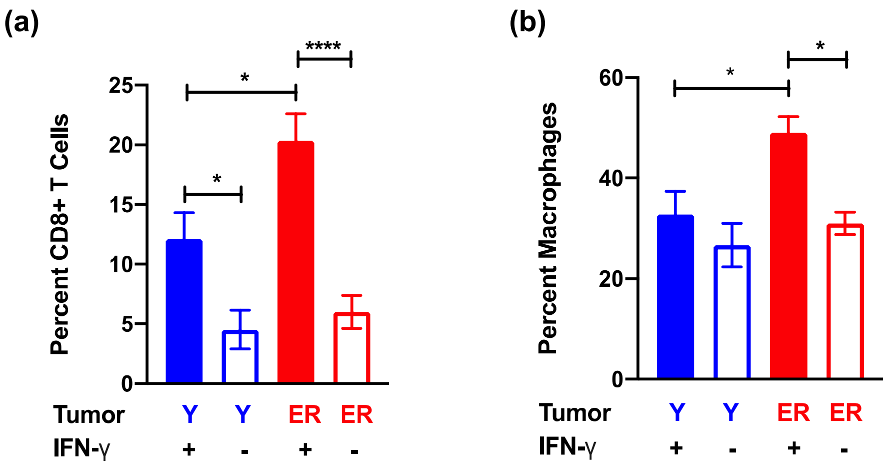

Figure S4: Percentage of tumor-infiltrating CD8+ T cells **(a)** and macrophages **(b)** in synchronous Y (ER + **Y**) and ER (**ER** + Y) tumors of wildtype and *Ifng*$^{-/-}$ mice on day 27. Data (mean ± SEM) are pooled from 3 - 5 mice/group/experiment and representative of at least two independent experiments *p < 0.05, **** p < 0.0001.

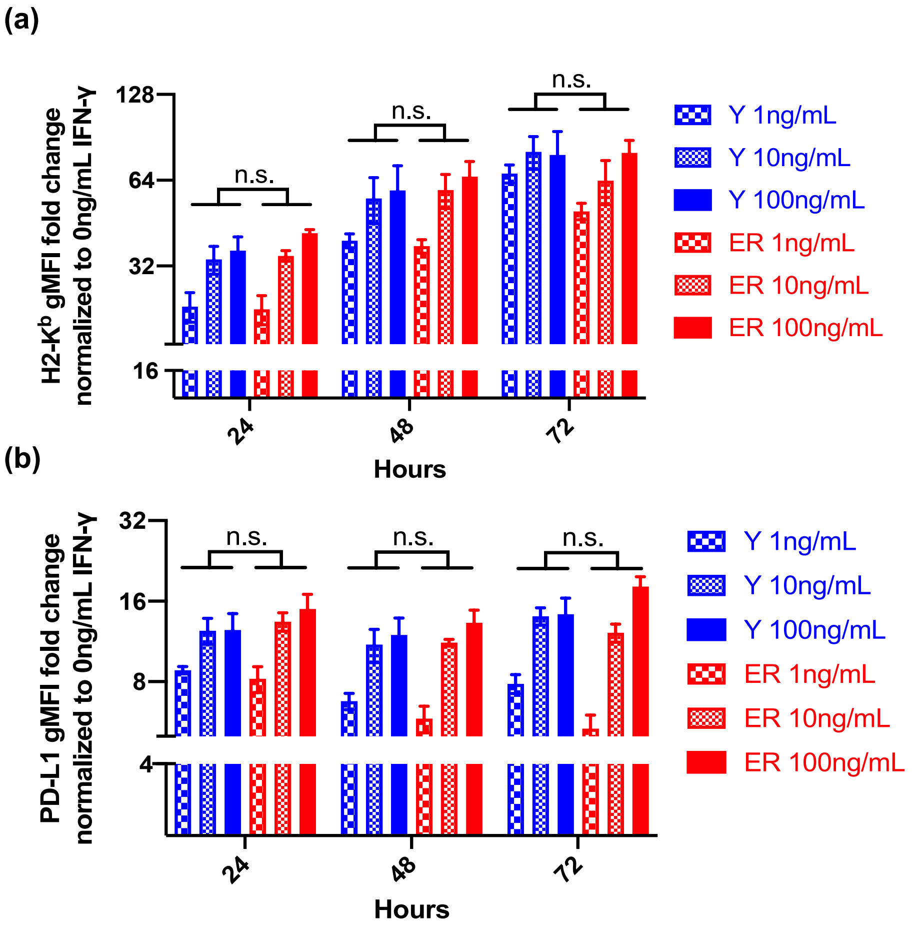

Figure S5: Normalized geometric MFI of surface H2-K^b **(a)** and PD-L1 **(b)** on YUMM (Y) and YUMMER (ER) cells cultured with exogenous IFN-γ in vitro. Data (mean ± SEM) pooled from 9 wells/sample from 3 independent experiments. n.s. not significant.
